# Heparin-Induced Hyperkalemia in anExtremely-Low-Birth-Weight Infant: A Case Report

**DOI:** 10.4274/jcrpe.1255

**Published:** 2014-06-05

**Authors:** Tomoyuki Shimokaze, Kazuhiro Akaba, Emi Saito

**Affiliations:** 1 Yamagata Saisei Hospital, Department of Pediatrics, Yamagata, Japan

**Keywords:** Aldosterone, dalteparin

## CASE REPORT

Heparin may cause hyperkalemia by blocking aldosterone biosynthesis in the adrenal gland. Dizygotic twin sisters were born by Cesarean section at 25 weeks’ gestation. The younger sister developed acute hyperkalemia (7.4 mEq/L) at 10 days of age. At the time of the development of the hyperkalemia, there were no signs of systemic infection, cardiac or renal failure, adrenal insufficiency, or sudden anemia. She was receiving no medication other than heparin to maintain the vascular catheter. Heparin was changed to dalteparin at 12 days of age. The plasma potassium level normalized after 14 days of age. After this change, the urinary potassium concentration and the aldosterone and plasma renin activity increased. The urinary aldosterone levels before and after the changes were 31 and 183 pg/μg creatinine, respectively. When heparin-induced hyperkalemia is suspected, stopping the heparin administration facilitates diagnosis and treatment; if anticoagulant therapy is required; one treatment option is changing from unfractionated heparin to low-molecular-weight heparin.

## INTRODUCTION

In neonatal intensive care unit, heparin is commonly used to maintain patency of vascular lines (1). However, heparin may lead to adverse effects such as bleeding, heparin-induced thrombocytopenia and osteoporosis. Hyperkalemia is also reported as a rare form of toxicity (2). Heparin may cause hyperkalemia by blocking the biosynthesis of aldosterone in the adrenal gland (3). Although there are reports of heparin-induced hyperkalemia in adults, there are no such reports in infants or in extremely-low-birth-weight (ELBW) infants.

We herein report an ELBW infant with heparin-induced hyperkalemia who showed improvement after changing unfractionated heparin (UFH) to low-molecular-weight heparin (LMWH).

## CASE REPORT

Our patient was one of the dizygotic twin sisters who were delivered by emergency Cesarean section at 25 weeks +5 days of gestation. There was no family history of multiple pregnancies. Magnesium sulfate and ritodrine administration did not achieve tocolysis, resulting in premature rupture of membranes of the older sister. Both infants were intubated and admitted to our neonatal intensive care unit. They underwent placement of a peripherally inserted central catheter (PICC) and arterial line. Intravenous fluid therapy with the addition of heparin at a concentration of 1.0 and 2.0 U/mL, respectively for PICC and arterial line, was begun to maintain the patency of the lines.

Our patient who was the smaller of the twins weighed 585 g at birth. She developed early nonoliguric hyperkalemia and dextrose and insulin infusion (maximal at 1 U/kg/day) was administered from 8 to 30 hours after birth. Intravenous administration of indomethacin was infused at a dose of 0.1 mg/kg for 1 hour at 14, 34 and 60 hours after birth to treat her patent ductus arteriosus (PDA). Closure of the PDA was confirmed by echocardiography at 4 days of age. Capillary heel blood samples were obtained several times every day and showed potassium (K+) levels of 4.0 to 5.7 mEq/L. The potassium level showed an increase at 9 days of age and rose to 7.9 mEq/L at 10 days of age.

The patient had no signs of asphyxia. Until 10 days of age, the infusion therapy including heparin at a dosage of 55 to 96 U/kg/day had been administered and K+ and sodium (Na+) intakes were 0 to 2.2 and 0 to 4.8 mEq/kg/day, respectively. At 10 days of age, her general condition was stable, blood pressure was 50/24 mmHg and diuresis level was 8.2 mL/kg/h. Her respiration had become relatively stable with the aid of a respirator and there were no signs of systemic infection, cardiac or renal failure, adrenal insufficiency or hemorrhage, intraventricular hemorrhage, or sudden anemia. She was given no medication other than heparin, indomethacin and enteral Lactobacillus casei. She was given breast milk and intravenous infusion therapy in amounts of 80 and 55 mL/kg/day, respectively. She was receiving heparin at a dosage of 55 U/kg/day. The electrolytes in the breast milk were measured several times and showed Na+ and K+ levels of 12 and 18 mEq/L, respectively. Laboratory test results before the insulin administration were as follows: arterial pH 7.35; pCO2 42.0 mmHg; HCO3- 23.1 mEq/L; white cell count 11.500/mm3; hemoglobin 15.0 g/dL; platelet count 140 000/mm3; lactate dehydrogenase 383 IU/L; creatine phosphokinase 144 IU/L; blood urea nitrogen (BUN) 6.0 mg/dL; creatinine (Cre) 0.8 mg/dL; cystatin C 1.82 mg/dL; magnesium 2.7 mg/dL; insulin 10.4 μIU/mL; and blood sugar 216 mg/dL. Urine collected by an indwelling catheter showed a pH of 8.0, osmolality of 90 mOsmol/kg, Na+ of 38 mEq/L, K+ of 4.9 mEq/L, Cre of 4.9 mg/dL, Beta-2 microglobulin (B2M) of 0.133/Cre, N-acetylbeta-d-glucosaminidase of 4.0 U/L, protein of 4.4 mg/day and no hematuria. The transtubular K+ concentration gradient was 7.8.

Only dextrose and insulin were administered to treat the hyperkalemia. Although the K+ in the intravenous infusion was removed, the total K+ was almost unchanged because of the continuous administration of breast milk (which contains K+) to maintain appropriate nutrition. Arterial blood samples were obtained several times a day and showed K+ levels of 6.1 to 6.6 mEq/L despite continuous insulin therapy at 1 U/kg/day.

The clinical and laboratory findings indicated that decreased K+ excretion without renal failure gave rise to the hyperkalemia. Heparin was suspected as the cause of her refractory hyperkalemia. At 12 days of age, heparin was changed to dalteparin. At 13 days of age, 24 hours after stopping the heparin, her blood K+ was 4.7 mEq/L under continuous insulin therapy at 1 U/kg/day and the insulin level was decreased gradually. Her blood K+ was 5.1 mEq/L under continuous insulin therapy at 0.5 U/kg/day from 48 hours after the discontinuation of heparin and the insulin was then also discontinued. At 15 days of age, 72 hours after stopping the heparin, her blood K+ level was 4.5 mEq/L without insulin administration. After changing the heparin to dalteparin, the fractional excretion of K+, aldosterone and plasma renin activity increased. There were no obvious changes in the cortisol level. The clinical and biochemical findings of the patient and treatment (Rx) data are summarized in Table 1. 

After the episode of hyperkalemia described above, there was no recurrence of hyperkalemia and the patient was discharged in good condition at the approximate expected date of birth.

## DISCUSSION

We have reported a case of an ELBW infant with UFH-induced hyperkalemia which improved after changing to LMWH. 

It is essential to maintain the patency of vascular catheters when treating critically ill infants and heparin is commonly used for this purpose. However, the safety and suitable quantities of heparin for administration are unknown (1).

Aldosterone secretion is mainly controlled by the renin-angiotensin-aldosterone system (RAAS) and K+ and adrenocorticotropic plays a minor short-term role (4). Renin secretion is stimulated by decreased NaCl transport in the distal portion of the loop of Henle, decreased pressure within the renal afferent arterioles and sympathetic nervous system stimulation of renin-secreting cells via beta-1 adrenoreceptors (5). Plasma K+ directly stimulates aldosterone secretion and indirectly modifies it by activating the local RAAS (4). The plasma K+ level needs to increase by as little as 0.2 mEq/L to stimulate significant aldosterone secretion (6). 

Heparin-induced hyperkalemia due to hypoaldosteronism is infrequent but notable because it may lead to fatal complications (3). Heparin lowers the number and affinity of angiotensin II receptors in the adrenal zona glomerulosa, which results in decreased plasma and urinary aldosterone concentrations (2,7). 

Our infant showed improvement in her severe hyperkalemia after the change from UFH to LMWH. Only dextrose and insulin were administered to treat the hyperkalemia because of exclusion of factors such as fluid and Na+ loading, forced diuresis and corticosteroids, which may make the pathophysiology unclear. In this preterm infant, the hyperkalemia developed late, after the typical time for development of non-oliguric hyperkalemia had elapsed. Hyporeninemic hypoaldosteronism as a consequence of prostaglandin inhibition by indomethacin may be considered as a possible mechanism for the development of the hyperkalemia, since prostaglandins are potent stimuli for renin release (8). Prostaglandin inhibition accompanies decreased urinary output because of the decreased glomerular filtration ratio and renal blood flow (9). However, our patient had normal serum BUN and Cre concentrations, a normal urinary B2M level which is an indicator of acute kidney injury (10) and preservation of normal urine volume. Furthermore, the plasma half-life of intravenous indomethacin in preterm infants is about 20 hours (11). Our infant developed hyperkalemia more than 180 hours after the last indomethacin administration.

Among adults, the risk for the development of heparin-induced hyperkalemia is reportedly associated with basal impairment of the RAAS and renal function (12). Higher K+ absorption in the gut, lower K+ secretion/excretion in the kidney and immaturity of the mechanisms regulating intra/extracellular K+ distribution as features of preterm infants predispose to K+ storage (13). In addition to these underlying factors, K+ excretion seemed to be compromised by heparin-associated impaired aldosterone reactivity in the present case.

The urinary and serum aldosterone levels increased after changing from UFH to LMWH. Only a slight increase in the plasma K+ level independently promoted aldosterone secretion in a K+-loading study of adults (6). No associations between spot plasma K+ levels and urinary or plasma aldosterone levels have been reported in infants (14,15,16,17). However, from these results, it is not clear whether normokalemia suppressed the plasma aldosterone or hypoaldosteronism caused the hyperkalemia. In neonates, because the K+ loading test is impossible, the influence of changing plasma K+ levels on aldosterone secretion is not clear. In our patient, we hypothesize that the aldosterone level did not increase in response to critical hyperkalemia. 

According to a previous review of heparin-induced hyperkalemia, renin activity remains constant or rises and is infrequently accompanied by hyponatremia (2). In our patient, plasma renin activity was lower during the hyperkalemic state. Reports have indicated a correlation between plasma renin activity and urinary Na+ excretion in neonates (14,16). If heparin suppresses aldosterone secretion, renin will be activated in response to Na+ loss and the renin reaction will vary according to the adequacy of the Na+ level. We suppose that the increased urinary Na+ continued in our infant and that plasma renin activity was gradually promoted.

Aldosterone suppression has been reported secondary to both LMWH and UFH (18,19,20). However, it is not clear which is associated with a higher risk because of the lack of randomized controlled studies comparing the frequency. However, one report showed that the incidence of hyperkalemia was lower with LMWH than with UFH at the doses currently used for prevention of thromboembolism (21). Our patient appeared to show a similar phenomenon when we switched from UFH to LMWH. Removal of the arterial catheter and PICC was considered, but since the cause of the life-threatening hyperkalemia was not apparent at that point and it was necessary to maintain the patency of the vascular route to treat the hyperkalemia, it was decided to continue the IV infusion. No anticoagulants are available for safe use in neonates other than UFH or LMWH (22). One report showed that in adults, fludrocortisones used to antagonize the hypoaldosterone state is an alternative therapy for patients with heparin-induced hyperkalemia when anticoagulant therapy is required (23).

In summary, our ELBW patient developed UFH-induced hyperkalemia, a finding which shows the necessity of monitoring the K+ levels during administration of heparin in infants. Measurement of urinary aldosterone level is useful to establish a differential diagnosis of the cause of hyperkalemia and to prevent anemia by frequent blood draws. When heparin-induced hyperkalemia is suspected, stopping the heparin administration will facilitate the diagnosis. However, when anticoagulant therapy needs to be continued, changing from UFH to LMWH and use of fludrocortisone may be effective.

## Figures and Tables

**Table 1:**
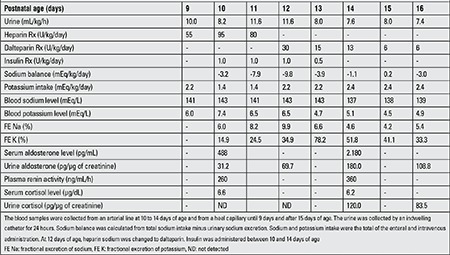
Clinical and biochemical findings of the patient and treatment (Rx) data
